# Construction Safety during Pandemics: Learning from the Xinjia Express Hotel Collapse during COVID-19 in China

**DOI:** 10.3390/ijerph182111498

**Published:** 2021-11-01

**Authors:** Yu-Jie Huang, Jing Tao, Fu-Qiang Yang, Chao Chen

**Affiliations:** 1 College of Environment and Safety Engineering, Fuzhou University, Fuzhou 350116, China; huangyujie199871@163.com; 2 School of Law, Fuzhou University, Fuzhou 350116, China; taojingruc@163.com; 3 Safety and Security Science Group, Faculty of Technology, Policy and Management, Delft University of Technology, 2628 BX Delft, The Netherlands

**Keywords:** construction accidents, COVID-19, STAMP, safety management, accident analysis

## Abstract

Many construction accidents occur in China each year, leading to a large number of deaths, injures, and property losses. Due to the outbreak of COVID-19, little attention is paid to construction safety, resulting in severe accidents. To prevent construction accidents and learn to how address safety issues in future pandemics, this study proposed an improved STAMP (Systems Theoretic Accident Modeling and Processes) model to analyze the collapse accident of the Xinjia Express Hotel used for COVID-19 quarantine in China. Through the application of the STAMP approach, the causes of the construction accident and the relationship between various causal factors are analyzed from a systematic perspective. The identified causes are divided into five categories: contractors, management of organizations, technical methods, participants, and interactive feedback. Finally, safety recommendations are drawn from this study to improve construction safety and safety management in pandemics.

## 1. Introduction

It is well-known that construction is a high-risk industry due to the complexity of the construction and the strategic effects of its products [1,2]. The accident rate in construction is an international concern [3], attracting increasing attention in academic and technical areas, as well as from the public. According to the statistical analysis report released by the Ministry of Housing and Urban–Rural Development in 2019, there were 773 production safety accidents in house and municipal road projects across the country, with 904 deaths as well as a year-on-year growth rate of 39 and 64 deaths. There were increases of 5.31% and 7.62% in 2018 [4,5]. Judging from the number of work safety accidents and fatalities in residential and municipal road projects across the country, safe construction should be further strengthened.

With the rapid development of China’s economic development, one of the key goals and tasks during the “14th Five-Year Plan” (2021–2025) period is to improve the level of urbanization [6]. Correspondingly, the total number of projects in my country is also rapidly increasing, causing many safety problems. Currently, analyses on the nationwide accidents related to transportation, hazardous materials, coal mining, and occupation have been conducted in China [7,8,9,10]. However, little research on construction industry safety has been carried out to depict the overall situation in recent years. Moreover, the current pandemic of COVID-19 leads to significant global social and economic disruption [11], resulting in new challenges for safety management and supervision. Many construction accidents occurred during the pandemic of COVID-19. Unfortunately, there is very little research on how to prevent construction accidents in China and how to prevent safety issues in similar pandemics in the future.

Although the Chinese central government and local governments already have promulgated a series of laws and regulations, and adopted many countermeasures to improve the safety of construction in our country, many accidents still occur in China each year. The improvement of project construction cannot be ignored. Especially in the period of COVID-19 and with similar public health emergencies, once a construction accident occurs, emergency rescue and follow-up projects will be more complicated. Considering that the spread of COVID-19 is mainly through human interaction, the interaction between rescue workers and trapped workers will play a leading role in the entire rescue process [12]. Recently, for instance, on 29 August 2020, a collapse occurred at the Shanxi Juxian Hotel, China, resulting in 29 deaths, 28 injuries, and direct economic losses of CNY 11.6435 million [13]. According to incomplete statistics, more than 30 large construction accidents in China occurred from January to September 2020 during the COVID-19 period [14]. With the spread of COVID-19 in countries around the world, recent scientific research has gradually been reported, involving discussions on how to improve the safety of COVID-19 [15,16].

It is important to use a system analysis approach to analyze the causes of construction accidents and to explore construction safety management during pandemics. Traditional accident models are based on fault models, such as the Domino accident model [17,18] and the Swiss cheese accident model [19]. They do not analyze the root cause of the accident from the perspective of system thinking [20] and assume that the accident is caused by the linear event chain. Although humans are recognized as an important factor in system failure, the assumption that human factor is an independent factor is generally not sound. Thus, it is difficult to explain the causes and solve the problem. 

In 2004, the accident causation model, the System Theoretic Accident Model, and Processes (STAMP) were introduced by Leveson (2004). According to the relevant literature, researchers use different analysis methods such as the Fault Tree Analysis (FTA), system thinking, the Human Reliability Analysis (HRA), Monte Carlo Simulation (MCS), cognitive reliability, the Error Analysis Method (CREAM), and the Formal Safety Assessment (FSA). However, these accident analysis methods were developed many years ago and have limited capabilities of analyzing complex systems. It is observed from the relevant literature that, despite its systematic and holistic analysis capability, the STAMP has not been properly applied in the construction field.

To explore the factors that contribute to construction accidents and to identify the solutions for accidents in construction, many researchers investigated the problem in different countries or regions [21,22,23,24,25]. Learning and researching from an accident is always a basic approach in accident prevention. As such, this paper aims to apply the STAMP approach to analyze a typical construction accident during the outbreak of COVID-19. The causes of the accidents related to safety management, migrant workers, and COVID-19 were analyzed. Additionally, safety recommendations to prevent construction accidents during pandemics are also discussed.

## 2. An Overview of Construction Accidents in China

**Figure 1:**
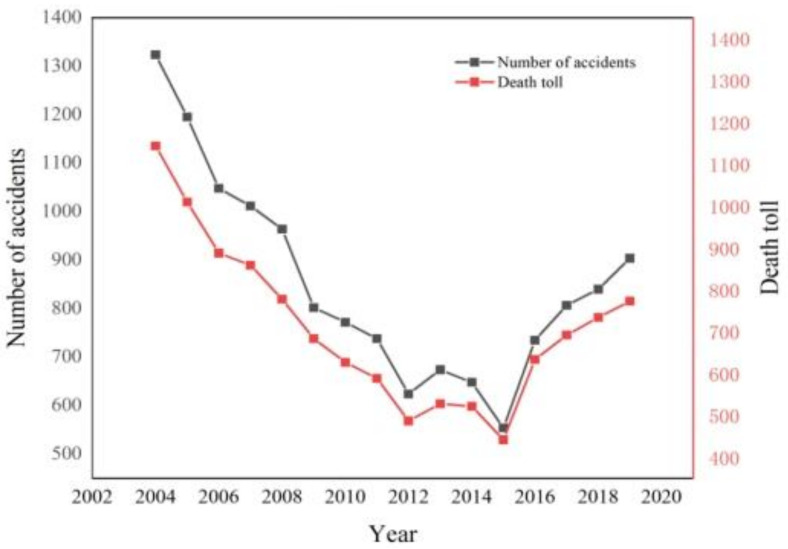
The number of accidents and death toll in construction in China from 2004 to 2019.

According to the statistics of construction accidents (MOHURD), the number of accidents and deaths related to construction in China is shown in Figure 1. It can be found that there was a decline in the frequency of accidents and the number of death tolls from 2004 to 2015. The improvement may be attributed to the safety measures taken by China, such as establishing the corresponding emergency mechanism and setting up the corresponding emergency department. In recent years, accidents in the construction industry have attracted increasing concern from the Chinese government, local governments, and companies. However, the number of accidents and fatalities gradually increased since 2015, mostly due to safety management issues. For instance, the contractor’s behavior in safety management has received increasing attention, including concerning the lack of personal protective equipment, regular safety meetings, and safety training [25].

**Figure 2:**
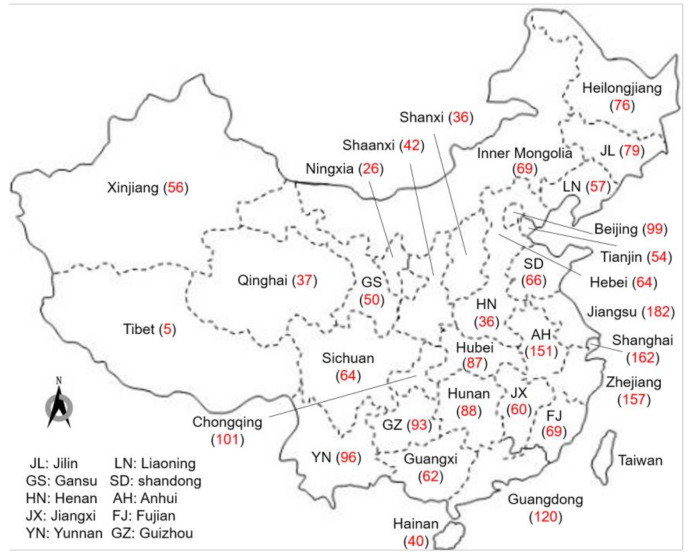
The geographic distribution of construction accidents in provinces (2015–2019).

The geographic distribution of construction accidents in each province from 2015 to 2019 is shown in Figure 2. Firstly, the total number of accidents in some areas is larger, such as in Jiangsu Province (182 construction accidents), Zhejiang Province (162 construction accidents), and Shanghai (157 construction accidents). They are in coastal cities and industry is more developed compared to other inland provinces and cities, therefore there are naturally many construction projects, which in turn leads to an increase in the number of accidents and fatalities. Secondly, the number of fatalities in some areas rose higher than in the same period last year. For example, the number of deaths in Sichuan, Anhui, Hebei, Xinjiang, and other regions rose by more than 50% year-on-year. Thirdly, mass fatalities and injuries had not been effectively curbed. In particular, the Hebei Hengshui “25 April” construction hoist fall (11 people died) and Shanghai Changning “16 May” plant collapse (12 people died), which represent two major safety accidents, resulted in heavy casualties and extremely bad impacts. The construction industry was one of the first industries affected by COVID-19 [26] and has experienced a high rate of infection [27].

## 3. Materials and Methods

STAMP was proposed in 2004 by Leveson to capture more types of accident cause factors, including the social organization structure, new human errors, design requirement defects, and poor cross-linking between non-faulty components [28]. STAMP is different from the previous safety analysis methods [29]. The analysis method is based on system theory and control theory, and considers safety as the emergence of complex systems [30]. According to the STAMP model, accidents do not occur due to component failure but rather they appear because of inadequate control and ineffective safety constraints.

Initially, professor Leveson applied the STAMP model to the “Columbia” crash accident and “Challenger” explosion accident [31,32], and achieved good results in the cause analysis. After that, the Leveson team and other researchers applied the STAMP model to various fields. For example, Leveson and Fleming analyzed aviation accidents [33]. Dekker and Couturier applied the STAMP model to medical malpractice [34]. Professor Grabowski and his team are applying it to oil and gas process safety [35]. Additionally, many domestic scholars have applied the STAMP model to the analysis of railway accidents and coal mine accidents.

**Figure 3:**
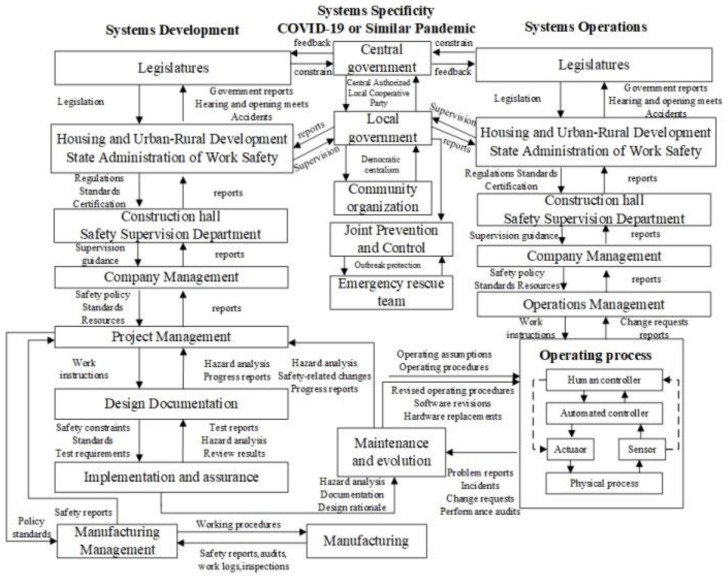
The hierarchical safety control structure of the improved STAMP combined with COVID-19.

The improved STAMP model considers the complex system as a hierarchical structure with multiple levels (see Figure 3). The improved model in Figure 3 has two basic hierarchical control structures—one for system development (on the left) and one for system operation (on the right)—with interactions between them. However, this article combines the characteristics of COVID-19 and adds a middle control structure. On this basis, a new control structure called system specificity is added. Figure 3 shows the hierarchical safety control structure of the improved STAMP combined with COVID-19. In this way, the improved STAMP model is generally divided into three parts, including system development, system specificity, and system operation. The topmost of this control structure has feedback and constraints. The entire system implements the constraint control from the top to the upper part, thereby achieving the normal operation of the system. When the system has invalidated the components due to certain external factors, the operation will occur within the system and the accident will occur.

According to Figure 3, it can be seen that the State Council, the Ministry of Housing and Urban–Rural Development, and the State Administration of Work Safety control the overall safety management of the construction industry based on legislation and issued normative standards. The local government, the construction hall, and the safety supervision department formulate corresponding local regulations and directly control the development of various engineering projects within their jurisdiction. Each engineering project adopts the organizational form of the project department and all parties involved in the construction coordinate and restrain each other to control the actual progress of the engineering project. Frontline laborers accept the instructions and constraints of the construction unit. Then, they perform labor operations and provide feedback.

When COVID-19 or a similar pandemic occurs, the central government first makes professional decisions, scientific decisions, and issues instructions. At the same time, the central government is restricted by the legislatures and provides feedback to the legislatures. Then, the central government authorizes local cooperative parties and the local government both executes orders and subsequently centralizes resources. Finally, the community organization conducts a grid investigation. If a construction safety accident occurs during this period, the joint prevention and control department require the emergency rescue team to consider epidemic prevention. The model is constructed by using the relationship of the control structure to reveal the control requirements or constraints of the upper layer to the lower layer, as well as to feedback the information of the lower layer to the upper layer.

**Figure 4:**
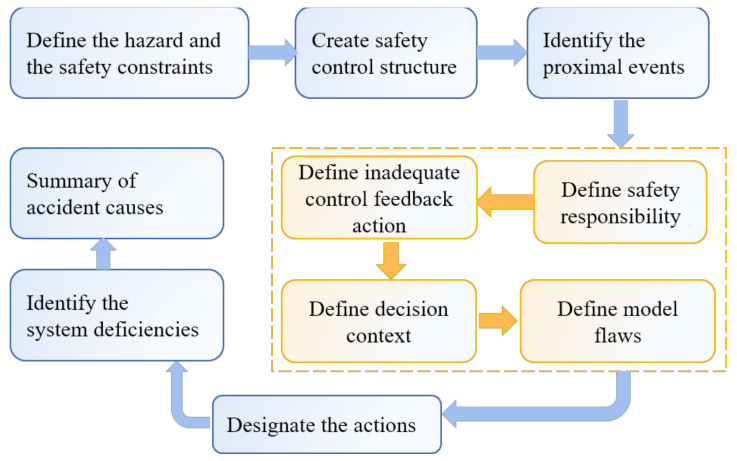
Flow chart of the method.

The process of applying STAMP to analyze the accident is divided into nine stages, which is defined by Leveson (2012) as CAST (Causal Analysis based on STAMP). It is a technique and process designed specifically for accident analysis. The CAST method draws a framework for applying the STAMP analysis to incident cases. Using the CAST method does not simply find the cause of the incident but rather it provides a holistic view of the incident and allows the entire system to be addressed, alongside implementing internal changes. To understand accidents by using the CAST method, we modified Leveson’s CAST steps as demonstrated in Figure 4. The analysis consists of the following application steps: (i) defining the hazard and safety constraints; (ii) creating the safety control structure of the accident; (iii) identifying the proximal events of the accident; (iv) defining some detailed contexts, such as safety responsibility, inadequate control feedback action, decision context, and model flaws; (v) designating the actions by considering the system structure; (vi) identifying the system deficiencies; and (vii) forming the summary of the accident causes.

## 4. Case Study

The Xinjia Hotel collapse accident that occurred in Quanzhou, Fujian Province, was selected as the analysis case study for a variety of reasons. The event is a special and major construction accident in China. According to the Regulations on the Reporting and Investigation of Production Safety Accidents of China, the accident, which killed 29 people, did not reach the level of particularly serious accidents. However, it was surveyed and handled by the State Council as a particularly serious accident. The upgraded investigation of the accident shows that the nature of the accident was serious and the impact was bad. In addition, at the time of the incident, the hotel was a centralized, isolated observation point for the prevention and control of COVID-19 in Licheng District, Quanzhou City. The collapse accident occurred in this special period, resulting in the complexity and difficulty of the emergency rescue, accident investigation, and disposal.

According to the new Ministry of Emergency Management (MEM) of our country and the research report on the collapse of the house hotel restaurant, the root cause of the safety accident is already known. The safety accident company changed the new house hotel restaurant building from the original four-story construction to a seven-story construction to achieve the ultimate load-bearing capacity, thus it was vulnerable to collapse. There was also illegal structural reinforcement and electric welding at the bottom support-point steel column before the accident, which caused the instability of the steel column and led to the overall collapse of the building.

**Figure 5:**
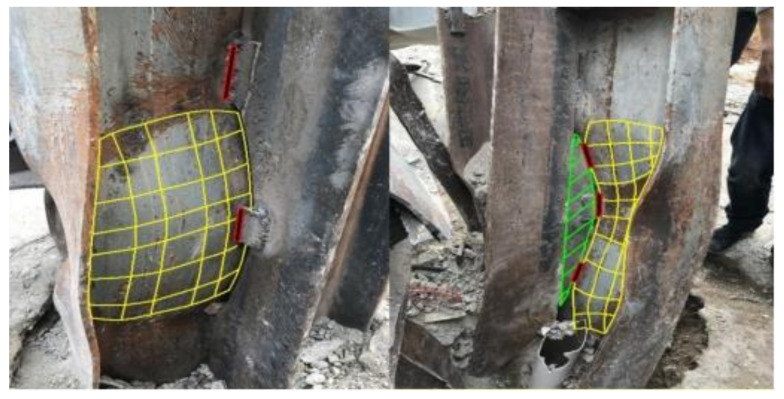
Buckling deformation and welding of C6 steel column. From the Investigation Report on the Collapse of Xinjia Hotel, Quanzhou City, Fujian Province.

The vertical ultimate bearing capacity of the original four-story steel structure of the building was 52,000 kilotons. However, the actual vertical load was 31,100 kilotons, which reached 60% of the ultimate bearing capacity of the system. After increasing the interlayer to seven stories, the actual vertical load of the building structure had risen to 52,100 kilotons, which exceeded its ultimate bearing capacity of 52,000 kilotons. Local buckling and yield damage occurred in some critical columns of the structure (see Figure 5). Therefore, the vertical load exceeded the limit and caused the collapse.

**Figure 6:**
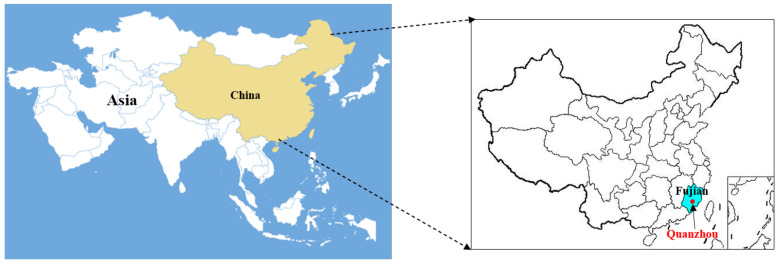
Location of the collapsed building.

The disturbance of the welding reinforcement operation caused the collapse. In welding reinforcement, the position of welding is not symmetrical and uniformity, weld length, and welding quantity are considerable qualities. The protection measures, such as unloading, were not taken. Figure 6 presents the positioning map of collapsed buildings. It is obvious that this collapse accident occurred in Quanzhou City, Fujian Province, China.

## 5. Results

### 5.1. Hazard and Safety Constraints

**Figure 7:**
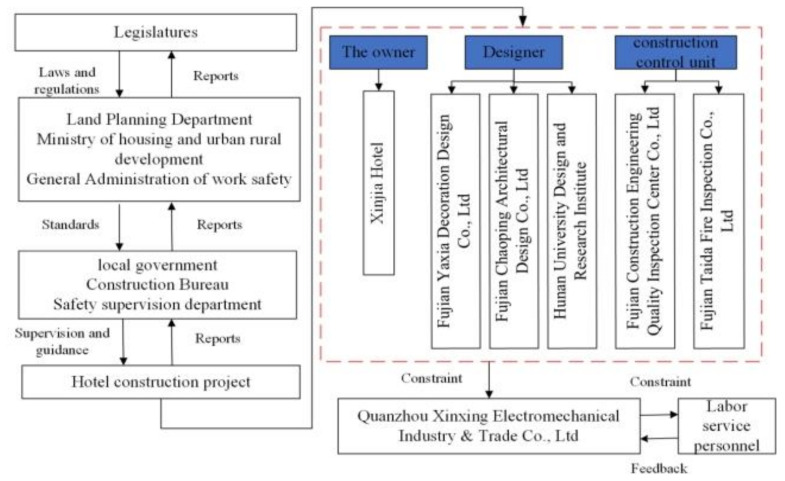
Safety control structure chart of the Xinjia Hotel construction project.

The Collapse Accident has a complex structure that contains the local government, construction bureau, safety supervision department, owner, designer, construction control unit, and labor service personal, among others, as shown in Figure 7. According to the studied accident case, the renovation of the building from four stories to seven stories is considered a hazard, which led to the collapse of the building because the vertical load exceeded the ultimate bearing capacity. Safety constraints prevent the construction of illegal buildings with the help of control-feedback and for the purpose of protecting the stability of buildings. Safety constraints and requirements related to hazards are divided into two system constraints: (1) physical point components must operate within limits and tolerances, and (2) maintenance and repair activities must correct any point defects. In addition to these, decision-making, communication, and situational awareness skills of the construction project are very important for the control structure.

### 5.2. Safety Control Structure

The second step of STAMP is to design the control structure, including control and feedback cycles. When the accident occurred, the hotel building was constrained by multiple control structures, which were responsible for ensuring the safety of the hotel building (see Figure 7). Figure 7 shows the safety control structure chart of the Xinjia Hotel construction project. In this section, a control structure was illustrated by using the data in the report of the accident. Controls and feedback are of great importance in the structure.

The State Council, Ministry of Housing and Urban–Rural Development, State Administration of Work Safety, and other departments control the safety management of the construction industry as a whole through legislation and standards. For instance, local governments and relevant departments formulate appropriate local regulations and directly control the development of projects within their jurisdiction; each project adopts the organization form of the project department and the parties involved in the project both coordinate and restrain each other to control the actual progress of the project; and the first-line labor service personnel accept the instructions and constraints of the construction unit, carry out the labor service operation in practice, and relay the information back into the process. The owner of this control structure was the Xinjia hotel and the designer was Fujian Yaxia Decoration Design Co., Ltd.; Fujian Chaoping Architectural Design Co., Ltd.; and Hunan University Design and Research Institute. The supervision unit included Fujian Taida Fire Inspection Co., Ltd., and Fujian Construction Engineering Quality Inspection Center Co., Ltd. The core of this control structure was the two control levels of the project management department and labor service personnel.

### 5.3. The Proximate Event Analysis

Proximate events of the collapse accident are determined in the third step of the application. The recent events leading to the accident are described according to the conditions and maintenance activities of the main points (see Table 1). The nearest incident to the accident is the weakening of the welded steel plate and the top floor plate. This corresponds to significant damage to the internal structural elements.

### 5.4. Responsibilities, Actions, Contexts, and Model Flaws

This step includes safety responsibility, control–feedback actions, the context of decisions, and model flaws of the owner, first controller, designer institute, construction control unit, land planning department, urban management department, housing and urban–rural construction department, and local party committee government, as shown in Table 2. The components within the control structure will be examined in detail.

### 5.5. Determination of Contributory Factors

The accident has not been analyzed with the chain of events method and a single component has not been blamed. With the help of STAMP, the collapse accident was evaluated holistically. Thirteen root causes were detected and are listed as shown in Table 3.

## 6. Discussion

To promote the concept of sustainable development in the construction industry, this article researched the construction industry, researched engineering construction safety production accidents in the past five years, and examined a recent classic case safety accident. Therefore, this section is dedicated to discussing construction safety in China. Especially when encountering a public health emergency similar to COVID-19, it is essential to know how to address construction safety incidents under these circumstances. The key findings of this study are discussed in this section.

### 6.1. Possible Causes of Construction Accidents Occuring in China

#### 6.1.1. The Large Scale of the Construction Industry in China Derived from Urbanization

Accordingly, the number of construction projects in China is higher and the size of a part of these projects is larger than in other countries. It only took a few decades for China’s construction industry to achieve its current scale of operations. The total value of my country’s construction industry is extremely large. Although the growth rate has slowed down to a certain extent in recent years, the very high total value and very low informatization management rate have prompted the construction and construction informatization management to greatly improve indoor space. Although the “safety first” standard has been included in the “Safety Production Law of the People’s Republic of China” since 2002, the potential safety hazards and failure rate remain serious.

#### 6.1.2. Weak Safety Awareness and Insufficient Safety Knowledge

According to the investigation report of construction accidents in the past five years, most of them were caused by human error. The result shows that many construction workers lack safety awareness and professional skills. Most of the practitioners in the construction industry in my country do not have high diplomas and do not understand the safety accidents that are likely to occur. Therefore, practitioners usually take a chance and ignore the prevention of safety accidents, sometimes causing serious safety accidents. In addition, construction engineering companies may not be able to adequately inform the contractors of the risks that may exist in the workplace of the employees and it is also likely that the lack of supervision of such employees may cause safety accidents.

#### 6.1.3. Insufficient Safety Management and Control of Dangerous Projects

According to the statistics, in 2018, various localities implemented key supervision and inspection services in sub-projects with greater risk. A total of 320,155 projects were inspected and a total of 11,302 illegal acts were investigated. In general, there were many violations of laws and regulations in construction projects. The implementation of such safety laws and regulations, policies and regulations, and manuals and norms, in particular, depend on safety supervision. After identifying the hazards, many construction accidents still occurred, which shows that Chinese construction companies need to strengthen safety supervision. If rectification is not carried out, the safety situation will not improve.

### 6.2. Possible Future Work Requirements of Construction Safety in China

Based on the findings of the above analysis, the corresponding proposal can be put forward.

#### 6.2.1. Promote the Continuous In-Depth Development of Special Rectification of Safety Production

We need to conscientiously implement the “Three-year Action Plan for the National Work Safety Special Improvement” and the “Three-year Action Plan for the Special Urban Construction Safety Improvement”. It is necessary to firmly advance various tasks and to promote the modernization of both the construction safety governance system and governance capabilities. Attaching great importance to the major safety risks of illegal construction and severely cracking down on behaviors that cause safety risks, such as illegal construction, illegal changes to the main structures of buildings, and illegal use of functions, will help to ensure safe practice.

#### 6.2.2. Understand Epidemic Prevention and Control the Normal Conditions of Construction Safety Work

We need to seriously study and judge the adverse impacts of the full resumption of the work and production of engineering projects on construction safety work. At the same time, we should implement the “Notice of the General Office of the Ministry of Housing and Urban-Rural Development on Strengthening the Prevention and Control of the COVID-19 and Promoting the Orderly Resumption of Work in Enterprises” and other document requirements. We must adhere to epidemic prevention as well as to control and construction safety measures. We must also prevent blindly initiating construction schedules resolutely and urge construction units to ensure reasonable schedule arrangements for construction projects to ensure construction safety.

### 6.3. The Limitations of This Work

This article selects a collapse accident during the COVID-19 era as an analysis object, which, in turn, extends to safety management and measures across the construction industry. This work is generally with certain limitations because its research range is more narrow. Additionally, the collapse accident that occurred in the special period of COVID-19 was selected as a case study, having certain specificity but not universality. If the conditions are allowed, it is best to analyze construction accidents that occur during different pandemics. In this way, when addressing all kinds of sudden epidemic situations, it will teach people how to initiate safety management and measures for building construction projects. In addition, this article only used the STAMP method to analyze the architectural accident. Future research studies can apply a variety of ways and models to analyze the reasons for architectural accidents and can propose measures from multiple perspectives.

## 7. Conclusions

The conventional accident analysis is focused on the chain of events, since it has limited ability to overcome non-linear interactions among dynamic and socio-technical system components. To overcome this limitation, a systematic accident analysis can be used to define the root causes of accidents from a holistic perspective. The STAMP method was used for the collapse accident for systematic accident analysis. The accident contributory factors were listed from a systematic approach and the result illustrates that STAMP treats accidents as complex systems rather than as simple chains of events. This study used the STAMP model and a case study of a construction accident that occurred during the COVID-19 period to explore construction safety management during epidemics. The following conclusions were obtained:

(1) A specific collapse accident case in the construction history is analyzed during COVID-19 as a demonstration of the STAMP method.

(2) In the present study, we used the relationship of the control structure to reveal the control requirements or constraints of the upper layer to the lower layer, and to feedback the information of the lower layer to the upper layer. When the accident occurred, the hotel building was constrained by multiple control structures, which were responsible for ensuring the safety of the hotel building. The core of this control structure is the two control levels, namely the project management department and the labor service personnel.

(3) From the analysis, it can be found that the recent events that led to the accident were identified as nine nodes, beginning from June 2012 at the earliest. The earliest event concerned the building of the new four-story steel structure in the plot. The nearest incident to the accident concerned the weakening of the welded steel plate and the top floor, which occurred on 7 March 2020.

(4) It can be considered as the first detailed implementation of the improved STAMP approach to building construction accident analyses during COVID-19. STAMP is a qualitative analysis. Therefore, system deficiencies were added to the analysis to fill this gap.

In providing a detailed STAMP application to the construction industry, the findings of the paper will contribute to construction safety management and will be helpful to both safety inspectors and the owners at the stage of the socio-technical investigation of construction accidents, analyses, and necessary measures’ implementation. Further research will concern the quantification stage of STAMP, which has yet to be applied to the construction industry as well as to others, since it can provide a proactive accident analysis tool to the construction field.

## Figures and Tables

**Table 1:** The proximal events leading to the hotel collapse accident.

Date	Events
July 2012	A new four-story steel structure building was built on the plot
May 2016	Mezzanine was added to the interior of the building from four floors to seven floors
July 2017	Hotel rooms on the fourth, fifth, and sixth floors were renovated
10 January 2020	It was found that the flange and web of steel column were deformed seriously by decoration workers
10–15 January 2020	Yang found that the other two steel columns were also deformed, asked workers not to talk, and decided to stop decorating
January–March 2020	Affected by the Spring Festival holiday and epidemic situation, no reinforcement construction was carried out
1 March 2020	Three steel columns deformed
5 March 2020	Welding operations were began
7 March 2020	The welded steel plate was not tightly adhered to the top floor plate

**Table 2:** Responsibilities, Actions, Contexts, and Models Flaws of the Components.

	The Owner	Designer Institute	First Controller of the Hotel	Construction Control Unit	Land Planning Department	Urban Management Department	Housing and Urban–Rural Construction Department	Local Party Committee Government
Safety responsibility	Honesty to submit relevant materials and reflect the real situation to the administrative organ Apply for construction projects according to the law	Undertake projects in accordance with regulations The review is unqualified and not accepted	Responsible for the hotel operation Responsible for the hotel check Provide real materials and reflect the real situation Report problems to relevant departments in time	Carry out inspection, repair, and maintenance according to national and industrial standards Make objective, true, and complete records of service conditions	Transfer the right to use state-owned land of commercial nature in accordance with laws and regulations	Daily patrol Implement the house building decision under special circumstances Investigation and rectification of housing safety hazards and comprehensive treatment of “two violations”	Implement project quality responsibility Guide, supervise, and manage construction activities Improve the project quality management system Improve the construction safety supervision system	Strictly fulfill the responsibility of management Implementation of the safety production responsibility system Supervise and guide the work of “cracking down on non-compliance” and so on
Inadequate control–feedback action	Failed to notify and guide people to evacuate in a timely manner There were no valid emergency response measures	Undertook decoration construction in violation of regulations Unqualified to undertake drawing modification Failed to stop illegal activities in time	There was no feedback to relevant departments concerning the condition of the hidden danger Inadequate inspection It was not controlled and there was no valid emergency response measures	Failed to provide built drawings and design drawings of the fire construction unitOrganize the inspection of fire-fighting facilities and issue reports	Illegal transfer of land use rights and negligence	Failed to stop the illegal construction of the Xinjia hotel for many times	Failed to investigate and address long-standing violations of laws and regulations regarding construction No report to the party committee government, although it was required	Failed to understand and correct the investigation and governance Failed to make a scientific decision
Context of the decision	Time lag Lack of occupational experience Lack of responsibility The impact of COVID-19	No relevant qualification Time lag Lack of responsibility The impact of COVID-19	Time lag Age (65 years old) Lack of responsibility Weak safety awareness The impact of COVID-19	Time lag Lack of responsibility The impact of COVID-19	Time lag Lack of responsibility Normal bidding mode was not adopted	Planning license was not obtained Time lag Negligence in inspection The impact of COVID-19	Time lag Negligence in inspection Dizziness of responsibility consciousness The impact of COVID-19	Time lag Negligenve in inspection Dizziness of responsibility consciousness The impact of COVID-19
Model flaws	Failed to take emergency measures according to the law	Illegal acceptance and signature	Poor situation awareness concerning the building characteristics (he was unaware that it was necessary to stop the engineering to isolate the damage) Poor decision-making	Safety training was a mere formality	Establish a formality, go through the motions	Establish a formality, go through the motions	Long-term existence of lost checks and loss of guidance	There were serious problems of formalism and bureaucracy

**Table 3:** Contributory factors of the accident.

Sender	Receiver	Code	Description of the Code
Physical equipment	Labor service personnel	S1	Violation of reinforcement welding operation
Violation	First controller	S2	Inadequate inspection and ignoring of the danger
Violation	Increasing mezzanine	S3	Reached ultimate bearing capacity and in the collapse
First controller	Labor service personnel	S4	First controller asked workers not to speak
Land planning department	The owner	S5	Inadequate inspection and violation
Construction control unit	The owner	S6	No report concerning that the current detection failure could cannot be resolved
Party committee government	Construction control unit	S7	Unreasonable requirements
The owner	Designer institute	S8	Unreasonable restriction on the designer
External factor	Labor service personnel	S9	The emergence of COVID-19
External factor	Labor service personnel	S10	Time lag
External factor	Supervision unit	S11	Lack of contact
First controller	The owner	S12	Insufficient feedback to the company
First controller	The owner	S13	Insufficient procedures were not controlled, defined, and reported the company
